# Proposal for an Embedded System Architecture Using a GNDVI Algorithm to Support UAV-Based Agrochemical Spraying

**DOI:** 10.3390/s19245397

**Published:** 2019-12-07

**Authors:** Maik Basso, Diego Stocchero, Renato Ventura Bayan Henriques, André Luis Vian, Christian Bredemeier, Andréa Aparecida Konzen, Edison Pignaton de Freitas

**Affiliations:** 1 Electrical Engineering Graduate Program, Federal University of Rio Grande do Sul (UFRGS), 90035-007 Porto Alegre, Brazil; rventura@ece.ufrgs.br (R.V.B.H.); edison.pignaton@ufrgs.br (E.P.d.F.); 2 Institute of Informatics, Federal University of Rio Grande do Sul (UFRGS), 90035-007 Porto Alegre, Brazil; dstocchero@gmail.com; 3 Faculty of Agronomy, Department of Crop Science, Federal University of Rio Grande do Sul (UFRGS), 90035-007 Porto Alegre, Brazil; andre.vian@ufrgs.br (A.L.V.); bredemeier@ufrgs.br (C.B.); 4 Department of Computing, University of Santa Cruz do Sul (UNISC), 96815-900 Santa Cruz do Sul, Brazil; andreakon@gmail.com

**Keywords:** embedded image processing systems, UAV automated systems, precision agriculture applications, NDVI algorithm

## Abstract

An important area in precision agriculture is related to the efficient use of chemicals applied onto fields. Efforts have been made to diminish their use, aiming at cost reduction and fewer chemical residues in the final agricultural products. The use of unmanned aerial vehicles (UAVs) presents itself as an attractive and cheap alternative for spraying pesticides and fertilizers compared to conventional mass spraying performed by ordinary manned aircraft. Besides being cheaper than manned aircraft, small UAVs are capable of performing fine-grained instead of the mass spraying. Observing this improved method, this paper reports the design of an embedded real-time UAV spraying control system supported by onboard image processing. The proposal uses a normalized difference vegetation index (NDVI) algorithm to detect the exact locations in which the chemicals are needed. Using this information, the automated spraying control system performs punctual applications while the UAV navigates over the crops. The system architecture is designed to run on low-cost hardware, which demands an efficient NDVI algorithm. The experiments were conducted using Raspberry Pi 3 as the embedded hardware. First, experiments in a laboratory were conducted in which the algorithm was proved to be correct and efficient. Then, field tests in real conditions were conducted for validation purposes. These validation tests were performed in an agronomic research station with the Raspberry hardware integrated into a UAV flying over a field of crops. The average CPU usage was about 20% while memory consumption was about 70 MB for high definition images, with 4% CPU usage and 20.3 MB RAM being observed for low-resolution images. The average current measured to execute the proposed algorithm was 0.11 A. The obtained results prove that the proposed solution is efficient in terms of processing and energy consumption when used in embedded hardware and provides measurements which are coherent with the commercial GreenSeeker equipment.

## 1. Introduction

The use of unmanned aerial vehicles (UAVs) is becoming increasingly common for applying pesticides and fertilizers in agricultural areas [1]. Works in this area have focused on the use of UAVs in spraying these substances primarily due to their effectiveness, which implies a significant cost reduction compared to the conventional spraying method, as well as the reduced risks when compared to the use of manned aircraft. However, to allow the large scale usage of UAVs in precision agriculture, it is important to determine the most effective method for their autonomous navigation and control so that the desired precision for the intended agriculture applications can be achieved. To pursue this goal, it is necessary to conceive embedded algorithms that can guide the UAV navigation while employing their actuators so that they can autonomously follow the defined guidelines of any given mission.

Image processing is an important area in computer vision for studies related to the development of autonomous systems, which is also the case for emerging UAV technologies [2]. Through images captured by RGB or infrared cameras carried by a UAV, image processing algorithms can access the spatial domain of the pixel matrix which composes the image where the UAV is inserted. Consequently, it is possible to identify patterns or common elements on the images in real time [3,4,5]. These patterns allow for the extracting of data that can be used as the input in embedded autonomous decisions and control systems. In the case of UAVs, the flight and mission control systems [6] can use the output of the processed images to guide the UAV navigation and decide on the actuation in that environment [7]. This information can then be used to aid the decision-making process in the next steps of the flight or mission directions.

In precision agriculture, UAVs are used for different applications such as field mapping, images capture to construct a normalized difference vegetation index (NDVI) [8,9], monitoring plagues, spraying chemicals, and plant counting [10]. According to [11], NDVI is a mathematical index composed of spectral bands, recorded by sensors such as satellites, RGB, and infrared cameras. The work presented in [12] describes the use of NDVI and other indexes to monitor a maize crop by comparing the indexes acquired by measurements on the ground and those performed by a UAV. Considering the automated UAV for spraying pesticides and fertilizers, using the NDVI would be suitable to provide information to the navigation and mission control system to drive the UAV over the crops and deliver the chemicals according to the identified needs. However, the commercial-off-the-shelf (COTS) equipment used to acquire this index is hard to put on board of a UAV and interface with its navigation and mission control hardware and software. Therefore, the problem is how to design and efficiently deploy a real-time image processing and decision-making system that is capable of supporting the execution of an NDVI algorithm, possibly together with other image processing algorithms, and the mission control algorithm, in a UAV embedded computing platform.

A hyperspectral flying platform is presented in [13]. The solution is based on a commercial DJI Matrice 600 drone and a Specim FX10 hyperspectral camera. A Jetson TK1 board is used to control the drone trajectory, manage the data acquisition, and perform the onboard processing of different vegetation indices, such as the NDVI.

A system for estimating vegetation indexes in an area is proposed by [14]. This system consists of a small UAV performing autonomous flights and recording videos of the ground cover using a GoPro camera with a modified infrared filter lens for video acquisition.

The most challenging problem with image processing algorithms applied to UAV-based systems is mainly related to their excessive use of hardware resources, which is a crucial issue in real-time applications, such as the actuation control of a UAV, as discussed in [10,15,16]. Concerning the use of image processing results as the input to UAV control systems, frames per second (FPS) are usually an indicator to measure their efficiency [7]. The higher the number of frames processed per second, the faster the algorithm is. The challenge then is how to reach an FPS ratio that meets the algorithm requirements.

Observing this landscape, this work focuses on designing and developing a UAV embedded system to control chemicals spraying over crops using an index that provides the same type of information as the NDVI as input data, the green normalized difference vegetation index (GNDVI). This solution consists of a real-time image processing system using an adapted NDVI algorithm, the GNDVI. The adapted NDVI algorithm is deployed in low-cost hardware, a Raspberry Pi 3, and embedded in a small UAV for precision agriculture applications. The proposed system solution includes ensuring an efficient relationship between FPS and the speed of the UAV flight, thus requiring an efficient calculation of GNDVI levels while complying with the real-time requirements derived from this relationship. In addition, the solution is committed to the light-weight goal in terms of hardware resource consumption, so that it is possible to simultaneously run other image processing algorithms that could support other features of the automated design of a spraying UAV. Thus, the main contributions of this paper are (i) designing a complete system solution of autonomous control for a spraying UAV, and (ii) developing an embedded GNDVI algorithm that supports the real-time requirements of the control system.

The proposed system is based on low-cost hardware and able to perform index acquisition without requiring industrial level equipment, as in [13]. Although the use of more robust industrial equipment provides better system performance, this work demonstrates that the performance achieved by the proposed system is sufficient to meet the timing requirements of the application, even running on very low-cost hardware. In addition, there is no need to transmit data for processing on the ground as presented in [14]. Thus, the solution proposed in this work combines different approaches in a new embedded system proposal for autonomous control of a spraying UAV. A low-cost hardware architecture based on that in [7] was developed. Abstracting techniques from [15,16] allowed the mathematical optimization of the proposed image processing algorithm. Following [7,10], it was possible to abstract techniques for the use of an infrared camera, and from [4,10], techniques for image processing with illumination variations present in images used in precision agriculture applications.

This remainder of the paper is organized as follows: Section 2 presents an overview of the proposed system as well as its application scenario. Section 3 describes the proposed embedded GNDVI algorithm from the image acquisition, processing, and the resulting index. Section 4 describes the details of the software and hardware architecture developed in the project. Section 5 describes the designed experiments and the obtained results. Finally, Section 6 presents conclusions while providing directions for future work.

## 2. Proposed System Overview

**Figure 1:**
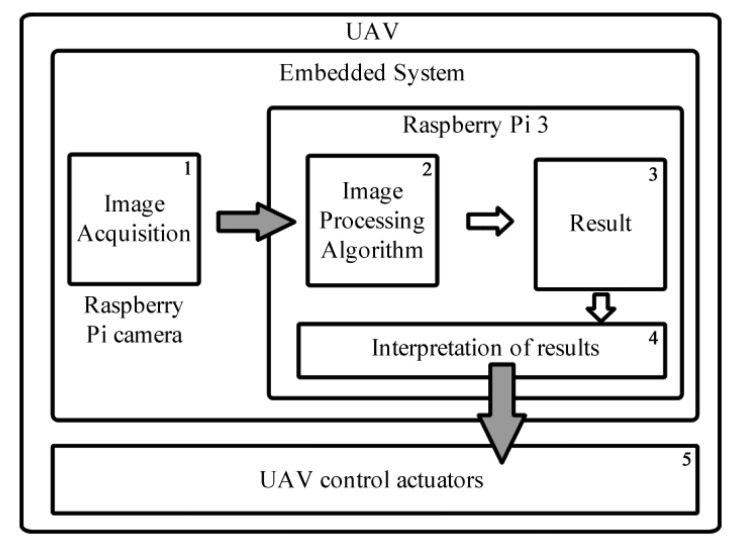
System design schematics.

The proposed system consists of a UAV actuator control software system based on data provided by an image processing algorithm. This software runs on embedded hardware integrated into the UAV platform, as shown in Figure 1. Despite the simplicity of the system operation, the high computational cost of image processing imposes a challenge to the low cost embedded processing hardware.

Following the schematic representation depicted in Figure 1, the video captured by the infrared camera (Number 1) is submitted to the image processing algorithm (Number 2) to determine the results for the timely application of the GNDVI for each pixel of the frame. Then, the average GNDVI for the current frame is calculated (Number 3). The embedded system uses this result as source data for decisions (Number 4), to control the UAV actuators (Number 5), and perform self-regulated applications of fertilizers or pesticides.

**Figure 2:**
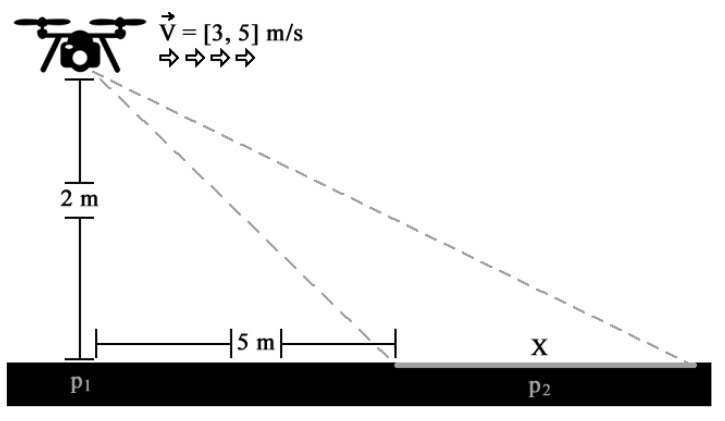
Application scenario for frame acquisition.

After acquiring the image, for example, a 1024 x 768 sized image, the average GNDVI is calculated for 786,432 pixels using the GNDVI algorithm. For each pixel, a sequence of subtraction, addition, and division operations is performed. In total, 2,359,296 mathematical operations are performed for each run; this done in a fraction of a second because of the current speed of available embedded processors, however, this is still a non-negligible time. Real-time applications often require more robust hardware with the use of powerful GPUs as demonstrated in [17]; however, it is not feasible to place such hardware in a small UAV due to the high cost and weight. In order to understand this issue, an application scenario for the operation of spraying UAV using the GNDVI algorithm is illustrated in Figure 2.

**Figure 3:**
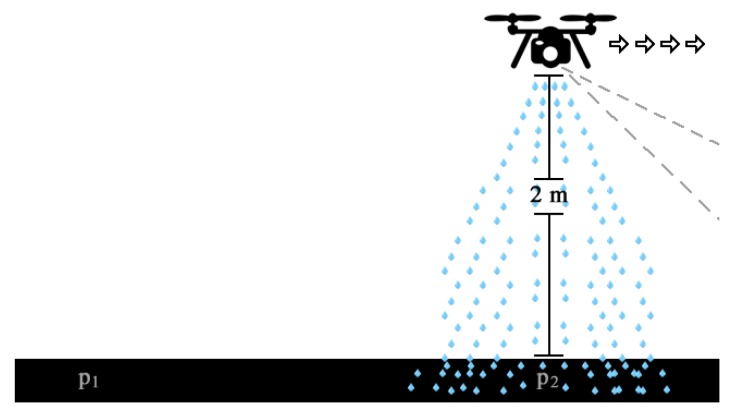
Application scenario for spraying.

In this scenario, the UAV flies at approximately 2 m above ground level while the camera focuses on an area 5 m ahead of the UAV position. The proposed system has been adjusted to work with this camera position to achieve the correct value of the reflection coefficient. In Figure 2, one of the dimensions of the area covered by the camera field of view is represented by *X*. Considering that the average speed of the UAV is between 3 m/s and 5 m/s, the minimum expected performance of the algorithm is greater than or equal to 1 FPS, so that the entire field is covered continuously without gaps. This number is obtained by dividing the distance of the UAV to the area currently covered by the camera field of view, by the speed of UAV displacement. Thus, the GNDVI results are processed and ready for use when the UAV passes over the area where the previous frame was captured, about one second earlier [15]. Figure 3 illustrates this situation, in which the UAV moved from point $p_{1}$ (the starting position in Figure 2) to $p_{2}$ (the center of the area covered by the camera about one second earlier) while spraying the fertilizers or pesticides over the area in $p_{2}$ following the algorithm decision.

## 3. The Proposed Embedded GNDVI Algorithm

The video captured by the infrared Raspberry Pi NoIR camera consists of sequential frames or images for emulating the movement sensation. Each captured frame is composed of the channels corresponding to the blue and green visible light spectrum as well as in the near infrared (NIR). The sensor captures the image obtaining the intensity of light for the respective spectrum channels at the same time *t*. The mathematical representation of the captured frame can be seen in (1).

(1) $$f=\left[\begin{array}{cccc} f(0,0) & f(1,0) & \cdots & f(W-1,0)\\ f(0,1) & f(1,1) & \cdots & f(W-1,1)\\ \vdots & \vdots & \ddots & \vdots \\ f(0,H-1) & f(1,H-1) & \cdots & f(W-1,H-1) \end{array}\right]$$

The captured frame is represented by a matrix with maximum width *W* and height *H*, and an origin in the upper left corner of the Cartesian plane. The function f(x,y) retrieves the pixel in the *x* and *y* coordinates, and *f* is the amplitude that determines the amount of light at that point in the digital image [3]. The pixel represented by (2) is the minimum entity that composes an image. In the infrared Raspberry Pi camera system, a pixel is represented by a vector $R^{3}$, where each position corresponds to the light intensity value in the NIR, green, and blue channels, respectively. Each channel is represented by 8 bits, where the value ranges from 0 the lowest to 255 the highest light intensity for that point.

(2) $$f\left(x,y\right)=\left[\begin{array}{c} NIR\\ G\\ B \end{array}\right]$$

The NDVI algorithm operates on the image in the spatial domain, with direct access to f while allowing for the differentiation of vegetation health, according to [18]. As shown in [11,19], the NDVI reflectance coefficient can be calculated from (3).

(3) $$NDVI=\frac{NIR-R}{NIR+R},$$

where NIR is the infrared and *R* the red channel values. Because the Raspberry Pi infrared camera does not capture the red channel, it is not possible to calculate (3). However, [11] demonstrated by validating two different datasets that GNDVI (Green NDVI) is a viable alternative to calculate the NDVI. The GNDVI and NDVI are strongly correlated [20] and can be used for any grain crop. Both are widely used for estimating several agronomic traits such as LAI in the common bean [21], as well as shoot biomass and chlorophyll and nitrogen content in wheat [22] and corn [23]. LAI is used in precision agriculture to control the application rate in sensor-based fungicide spraying in cereal crops in different locations within a given field [21]. The difference is that GNDVI uses the green instead of the red visible channel, as shown in (4).

(4) $$GNDVI=\frac{NIR-G}{NIR+G}$$

By applying (4), it is possible to obtain the GNDVI value for each pixel. The average GNDVI for each processed frame can be obtained by (5).

(5) $$GNDVI_{avg}=\frac{\sum_{x=0}^{W}\sum_{y=0}^{H}GNDVI\left(x,y\right)}{W\cdot H}$$

From the average GNDVI in (5), it is possible to estimate the application of pesticides and fertilizers. Although this calculation seems simple, the mathematical operations and algorithms need to be well organized and simplified to meet the real-time requirements, which are determined by the FPS rate and the UAV speed.

## 4. Implementation Details

### 4.1. Hardware Architecture

The hardware architecture is composed of an image processing system integrated into the UAV. This project used the UAV 3DR Iris+ quadrotor (3DR–3D Robotics, Berkeley, CA, USA) equipped with the Raspberry Pi 3 Model B image processing hardware and a 1.2 GHz 64-bit quad-core ARMv8 processor (Arm Holdings, Cambridge, UK), 1 GB of RAM and GPU VideoCore IV 3D graphics core (Broadcom Inc., San Jose, CA, USA). An SD card class 10 with 32 GB was used to store the operating system and the developed algorithms. The Raspberry Pi 3 (Raspberry Pi Foundation, Cambridge, UK) uses RAM as video memory, which was adjusted to 256 MB RAM to be used by the GPU.

The Raspberry Pi NOIR V1 Rev 1.3 camera (Raspberry Pi Foundation, Cambridge, UK) used for acquiring the videos has the same features as a common RGB camera, without the infrared filter that allows this channel to be captured. For the proper acquisition of infrared images, the blue gel filter [24] (Roscolux #2007 Storaro Blue) was fixed in front of the infrared camera. The blue gel filter together with the Pi Noir allows the health of green plants to be monitored [24], using the GNDVI and NDVI indexes.

The camera position was controlled by the attached gimbal Tarot T-2D V2 set to maintain the camera angle at 45${}^{\circ }$ to the ground, allowing a wider field of view and compensating for the FPS ratio and UAV movement speed.

**Figure 4:**
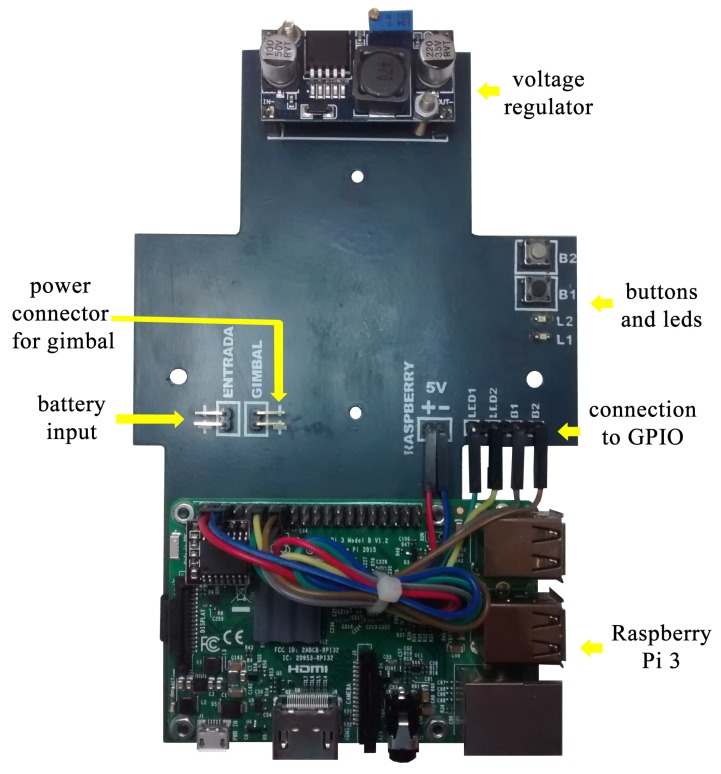
Developed integration board.

**Figure 5:**
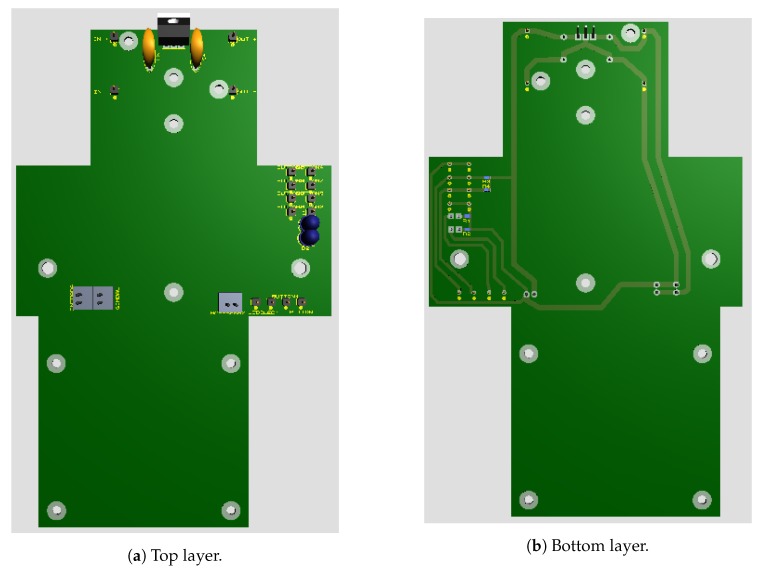
3D view of the developed integration board.

**Figure 6:**
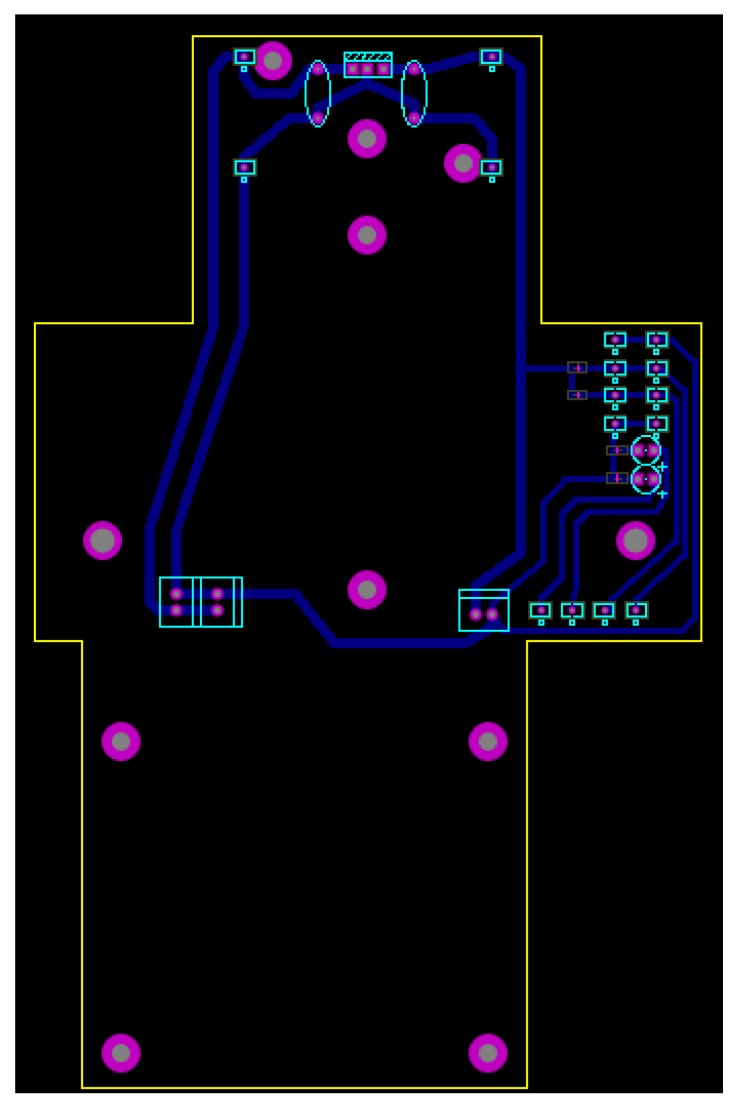
The bottom layer of the developed integration board.

**Figure 7:**
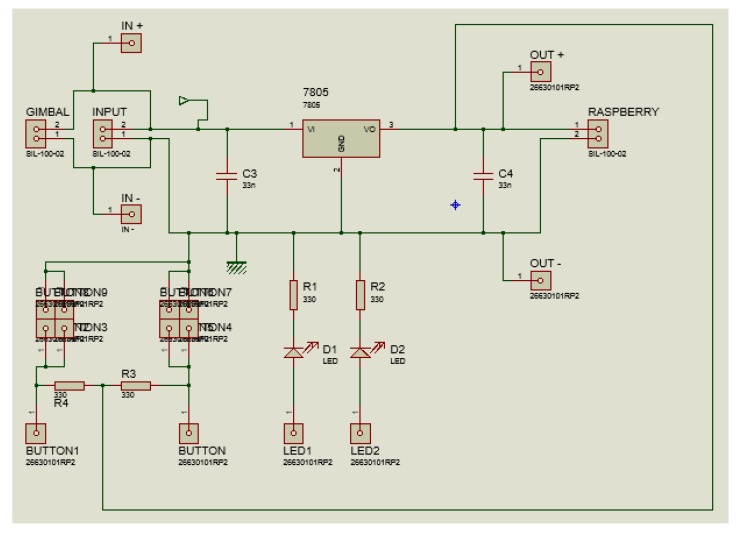
The schematic diagram of the developed integration board.

A board containing a voltage regulator circuit was built [25] for integrating all hardware and energy sources to the gimbal and Raspberry Pi. The board was installed between the UAV and the gimbal, allowing the Raspberry Pi to be fixed to the UAV body. Figure 4 shows the developed integration board for the embedded computing platform. The 3D view of the integration board can be seen in Figure 5. Figure 6 shows the bottom layer of the integration board. Finally, Figure 7 presents the schematic diagram of the proposed integration board.

**Figure 8:**
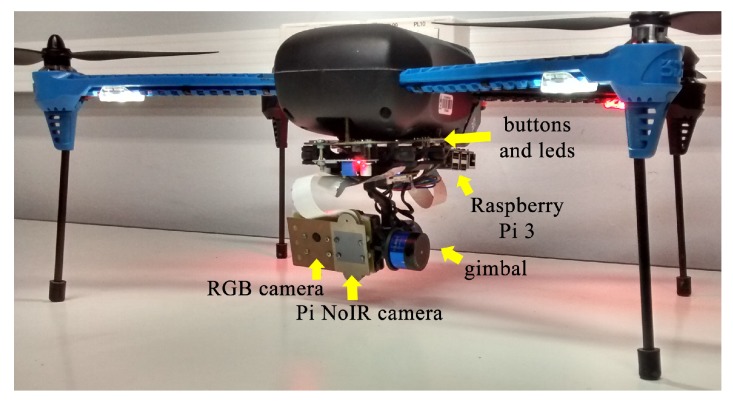
Final assembled hardware.

The camera cable was replaced with a long cable to allow the correct operation of the system with the gimbal. Two physical buttons and two LEDs were installed on the board and connected to the general purpose input/output (GPIO) of the Raspberry Pi to start the algorithm and for obtaining the system status, respectively. Figure 8 shows the developed hardware mounted on the 3DR Iris+ quadrotor.

The integrated hardware was tested and configured before installing and testing the algorithms. The RGB camera in Figure 8 was not used in this work; however, it was used for acquiring images used in other image processing algorithms running simultaneously in the embedded hardware, which was not the focus of this paper.

### 4.2. Software Architecture

**Figure 9:**
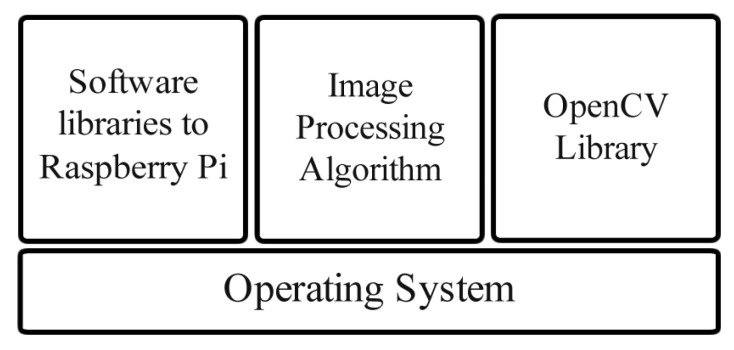
Software architecture.

The software architecture is divided into four modules as shown in Figure 9. The first module, in the bottom, is the operating system Raspbian Jessie [26] version 4.4 with disabled GUI interface for better performance. This operating system was chosen for compatibility reasons. The second module consists of the software libraries for the Raspberry Pi GPIO and Raspberry Pi camera which enables the control of the interface between the hardware and the developed algorithms. The third module is the developed image processing algorithm. The last module is the open source computer vision library (OpenCV) [27], used for image processing.

To test the proposed algorithm’s efficiency, two different applications were developed in two different programming languages: the first was Python and the second, C++. The implementation in both languages considered good coding practice for the performance, such as avoiding “for” structures and avoiding excessive parameters. The OpenCV library functions for mathematical operations on matrices were used to avoid the “for” structures. In addition, the OpenCV library was configured to use the GPU hardware via components of the open computing language (OpenCL) [28].

**Figure 10:**
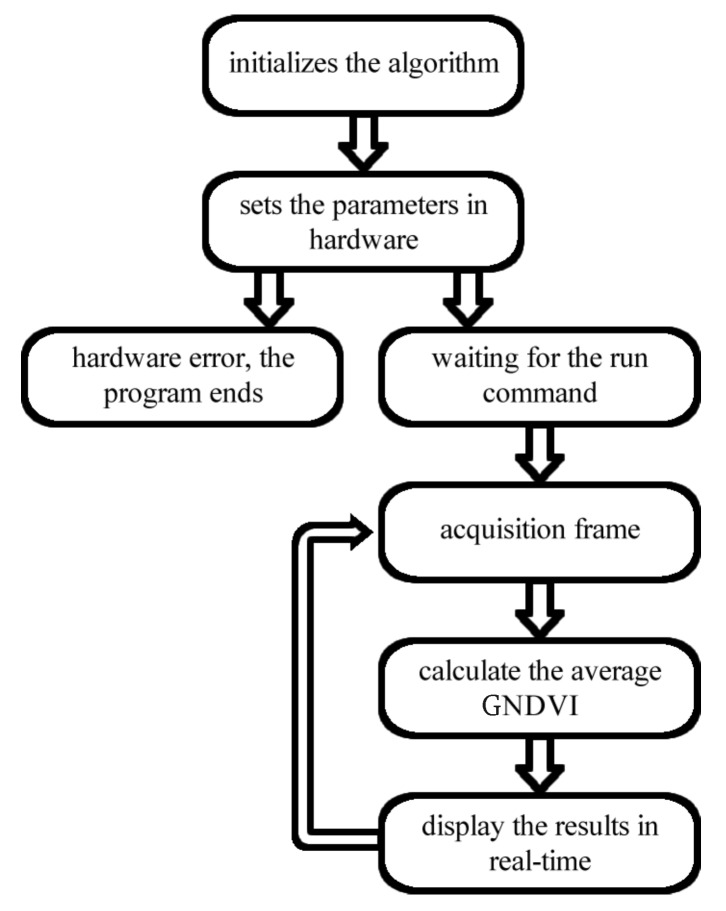
Algorithm flowchart.

The activity flow executed by the algorithm implemented in both languages is shown in Figure 10. It starts from the point in which the operating system (OS) is booted and all libraries are loaded. In addition, the implemented algorithm is configured for booting with the OS.

After starting the OS, the algorithm adjusts the appropriate settings in the Raspberry hardware and checks whether it is functional, otherwise, the algorithm is terminated, indicating an error. The configured hardware waits for a command to start, which is implemented by one of the buttons connected to the GPIO. After enabling execution, video frames begin to be captured following the preset parameters. The GNDVI is calculated for each frame using (5). The Raspberry Pi is also configured as a WiFi hotspot, making it possible to track the status of the algorithm and the average GNDVI in real time using a secure shell (ssh) connection in the Linux OS, made between the onboard Raspberry and a notebook or a smartphone.

## 5. Experiments and Results

Two types of experiments were conducted to prove the viability and correct functioning of the proposed system. The performed laboratory tests presented in Section 5.1 were followed in the field UAV tests, as shown in Section 5.2.

### 5.1. Laboratory Experiments

First, the performance of the developed algorithm was evaluated in the laboratory using the same hardware used in the field tests. Similar tests were conducted for implementing the proposed algorithm in Python and C++. The tests were conducted for nine different image resolutions acquired by the Raspberry Pi NoIR camera in real time. The highest resolution was 1920 × 1080 pixels (width × height) while the lowest was 133 × 100 pixels (width × height). In the performance tests, the measured variables were time to process a frame (TPF), in seconds; FPS; the percentage of CPU utilization; RAM usage, in MB; and virtual memory usage (SWAP).

To facilitate understanding, the results obtained in both experiments were separated according to the language implementation. The results for implementation using Python are seen in Table 1, while the results for implementation in C++ are seen in Table 2.

The performance analysis of both implementations of the proposed algorithm shows that both had low CPU utilization, about 20% for high definition (HD) image resolutions, and a low memory consumption, on average 70 MB. Additionally, the performance achieved with the C++ implementation was significantly higher, being up to 100% more efficient.

Considering the relationship between UAV speed and FPS, as detailed in Section 2, it is concluded that both implementations met the real-time requirements of the project while leaving available resources for the execution of other image processing algorithms that may be deployed in the system.

The resolution of the processed image does not interfere with the average GNDVI level for the same field area. Additionally, the average GNDVI can be captured using lower resolution images, thus improving the performance, and consequently, the available resources for implementing other algorithms, thus making the system more “intelligent” and/or able to perform other applications concurrently.

An additional experiment to collect voltage and current data was performed in the laboratory using a DC voltage and current meter equipment, to determine the energy efficiency of the proposed system. Twenty-two datasets were acquired in each run, at fixed intervals of 3 min.

The first run was performed with the proposed hardware whilst executing the operating system, but without the execution of the proposed algorithm. In the second run, the data were acquired with the same previous setup, but with the execution of the proposed algorithm using its implementation in the C++ language (Table 3).

It can be seen that the system uses an average of 0.26 A of electric current without executing the proposed algorithm, and 0.37 A when the algorithm is executed. On the basis of this information, the average current used to execute the proposed algorithm is 0.11 A. The average current of the whole embedded system (0.37 A) is less than or equivalent to the electrical current used by the fly control unit (FCU). Considering that the FCU has one of the lowest energy consumption rates of a UAV system, it is possible to state that the proposed embedded system has low consumption and can be used in small UAVs equipped with small batteries.

### 5.2. Field Experiments

**Figure 11:**
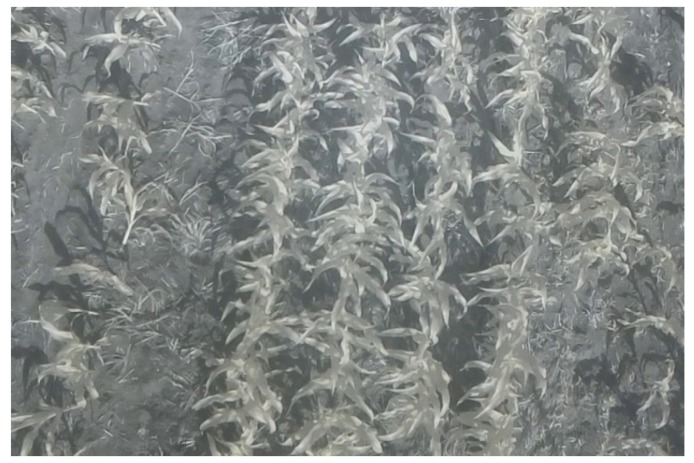
Infrared image captured by the Raspberry Pi NoIR camera in the test scenario.

The field tests were performed at the UFRGS agronomic research station (Eldorado do Sul, southern Brazil) on the 18 November 2016 at 15:40, in sunny conditions with few clouds. Figure 11 shows an infrared image captured by the Raspberry Pi NoIR camera during the UAV flight over the crop, following the scenario specifications in Section 2.

The test flights were over an experimental cornfield, with the algorithms running while the results were observed online via a connected notebook. GNDVI values were obtained at one point in the field for each image resolution, following the same procedures for both algorithm implementations. At the same time, readings were performed using the Trimble GreenSeeker handheld crop sensor [29], used in precision agriculture applications for determining NDVI.

**Figure 12:**
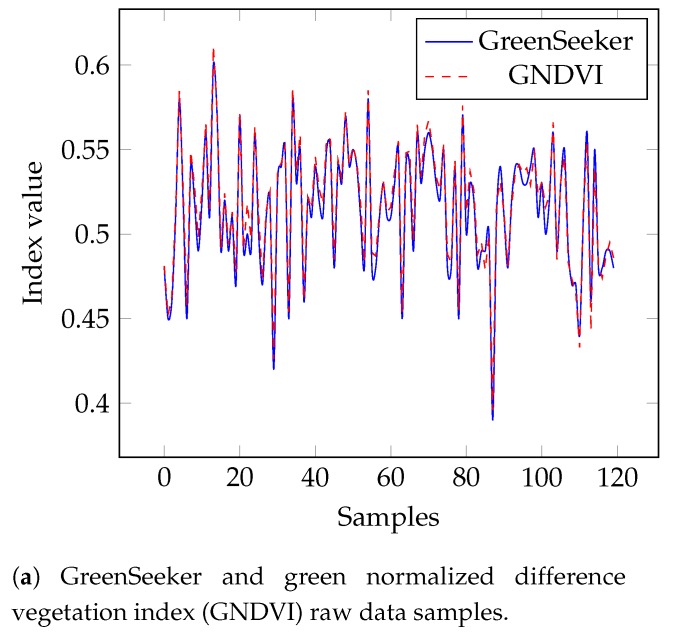

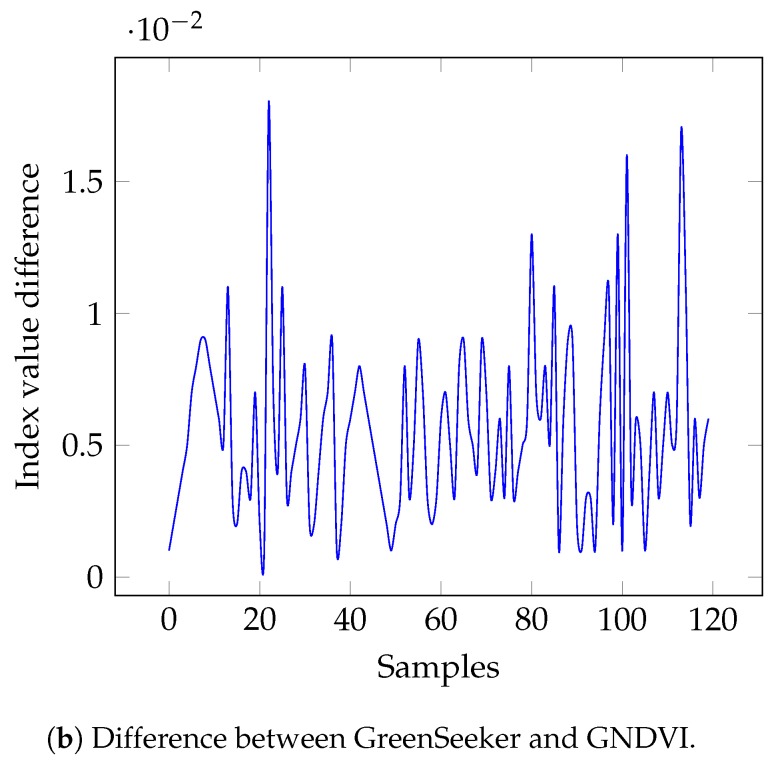
Comparison between normalized difference vegetation index (NDVI) acquired by the proposed embedded GNDVI algorithm and the GreenSeeker sensor.

The developed algorithm was validated by comparing the measured GNDVI values with those determined by the hand-held GreenSeeker sensor at the same time and location; this is because possible luminance differences could result in large variations. A total of 120 measurements were performed at the same points for the comparison, and the obtained results can be seen in the charts in Figure 12, in which Figure fig:samplesOfNDVIa presents the raw measurements and Figure fig:samplesOfNDVIb presents the difference between the values measured by the GNDVI and the GreenSeeker.

Whereas the GreenSeeker uses the red channel to calculate NDVI (3), the developed algorithm uses the green channel, as shown in (4), as this hardware resource is not available in Raspberry Pi NoIR camera. Therefore, a small variation in the achieved steady levels may be expected due to the different bands used, which was noticed as can be seen in Figure 12b. Regardless of the different setup, both indexes (GNDVI and NDVI) can be used to estimate crop health [11,20]. Indeed, Figure 12a shows that the two graphs almost overlap, highlighting the small difference between the GreenSeeker and the GNDVI results. The average difference between the GNDVI and GreenSeeker NDVI values was 0.0055 with a standard deviation of ±0.0033. The relative root-mean-square error (rRMSE) between the GNDVI and GreenSeeker NDVI values was ±1.24.

## 6. Conclusions

This paper reports the design and development of an embedded real-time system to support automatic agrochemical spraying using an onboard image processing algorithm. The complete design consists of a software system running in the embedded computing hardware integrated into a COTS UAV platform.

The performed tests showed that the implemented algorithms are computationally efficient, considering the real-time requirements of the applications, and effective, since similar values were obtained for the GNDVI and the NDVI determined by the GreenSeeker COTS sensor. The tests showed that the resolution of the processed image does not interfere with the average GNDVI obtained for each frame because the index is more strongly related to the proportionality of the elements composing the image than to its resolution. The use of lower resolutions can improve performance and consequently increase the available resources, allowing the simultaneous execution of other applications.

The next steps of this project are the implementation of a classifier algorithm capable of adjusting the actuators for the application of fertilizers and pesticides regulated by the developed algorithm. Additionally, a more detailed statistical study on sample classification for drying and wetting trends in fields could be performed to complement this work. Another direction for future work is the development of an algorithm to identify crop rows, in order to allow for a completely autonomous flight control for UAVs used in precision agriculture. 

## Figures and Tables

**Table 1:** Python results.

Resolution	TPF (s)	FPS	CPU (%)	RAM (MB)	SWAP (MB)	GNDVI
1920 × 1080	0.6	1.64	20	122	240.5	0.59
1336 × 768	0.29	3.4	18	65.7	179.5	0.61
1280 × 720	0.25	3.91	16	55.5	173.6	0.6
1024 × 768	0.24	4.1	15	49.5	127.8	0.59
800 × 600	0.16	5.99	12	46.9	168.1	0.57
640 × 480	0.12	7.82	11	41.9	164.8	0.56
320 × 240	0.083	12.03	7	39.1	160.1	0.55
160 × 120	0.075	13.29	5	37.1	159.6	0.54
133 × 100	0.021	45.98	4	36.8	159.4	0.59

**Table 2:** C++ results.

Resolution	TPF (s)	FPS	CPU (%)	RAM (MB)	SWAP (MB)	GNDVI
1920 × 1080	0.3	3.28	23	65.7	132.3	0.61
1336 × 768	0.15	6.59	22	39.6	111.3	0.6
1280 × 720	0.14	6.8	20	30.3	101.2	0.58
1024 × 768	0.12	8.29	21	30.5	99.7	0.578
800 × 600	0.083	12.021	17	28.7	97.6	0.58
640 × 480	0.059	16.92	17	24.6	94.5	0.58
320 × 240	0.024	41.51	9	21.7	91.5	0.59
160 × 120	0.023	43.028	5	20.2	90.9	0.6
133 × 100	0.022	44.21	4	20.3	91.1	0.59

**Table 3:** Energy efficiency experiment results.

Sample Number	Without Algorithm		With Algorithm	
	Voltage (V)	Electric Current (A)	Voltage (V)	Electric Current (A)
1	5.33	0.25	5.30	0.39
2	5.32	0.26	5.31	0.38
3	5.33	0.25	5.26	0.38
4	5.34	0.25	5.28	0.37
5	5.36	0.24	5.25	0.38
6	5.35	0.25	5.32	0.37
7	5.34	0.25	5.31	0.37
8	5.34	0.25	5.39	0.38
9	5.36	0.27	5.38	0.39
10	5.34	0.25	5.37	0.37
11	5.34	0.25	5.34	0.37
12	5.34	0.25	5.37	0.37
13	5.35	0.25	5.42	0.37
14	5.36	0.24	5.36	0.39
15	5.37	0.25	5.34	0.38
16	5.36	0.25	5.34	0.36
17	5.33	0.25	5.34	0.36
18	5.33	0.25	5.32	0.37
19	5.32	0.25	5.36	0.36
20	5.33	0.25	5.34	0.37
21	5.32	0.25	5.34	0.37
22	5.32	0.25	5.36	0.38
**Average**	**5.34**	**0.26**	**5.34**	**0.37**
